# Defects in the acid phosphatase ACPT cause recessive hypoplastic amelogenesis imperfecta

**DOI:** 10.1038/ejhg.2017.79

**Published:** 2017-05-17

**Authors:** Claire EL Smith, Laura LE Whitehouse, James A Poulter, Steven J Brookes, Peter F Day, Francesca Soldani, Jennifer Kirkham, Chris F Inglehearn, Alan J Mighell

**Affiliations:** 1 Department of Oral Biology, School of Dentistry, St James’s University Hospital, University of Leeds, Leeds, UK; 2 Leeds Institute of Biomedical and Clinical Sciences, St. James's University Hospital, University of Leeds, Leeds, UK; 3 School of Dentistry, University of Leeds, Leeds, UK; 4 Bradford District Care NHS Foundation Trust, Community Dental Service, Horton Park Health Centre, Bradford, UK

## Abstract

We identified two homozygous missense variants (c.428C>T, p.(T143M) and c.746C>T, p.(P249L)) in *ACPT*, the gene encoding acid phosphatase, testicular, which segregates with hypoplastic amelogenesis imperfecta in two unrelated families. ACPT is reported to play a role in odontoblast differentiation and mineralisation by supplying phosphate during dentine formation. Analysis by computerised tomography and scanning electron microscopy of a primary molar tooth from an individual homozygous for the c.746C>T variant revealed an enamel layer that was hypoplastic, but mineralised with prismatic architecture. These findings implicate variants in *ACPT* as a cause of early failure of amelogenesis during the secretory phase.

## Introduction

Amelogenesis, the formation of enamel, involves the secretion of a proteinaceous enamel matrix by ameloblasts. Progressive mineralisation follows through a series of repeated, cyclical processes that break down and remove matrix protein while simultaneously facilitating the growth of calcium hydroxyapatite crystallites.^1^ As the protein matrix is degraded, the space left is filled with expanding calcium hydroxyapatite crystallites, which forms highly ordered interlocking prismatic structures. This results in enamel that is over 95% mineral by weight,^1^ much higher than dentine (70%) or bone (65%).^2^

Amelogenesis imperfecta (AI) describes a group of inherited conditions characterised by defective amelogenesis, with a prevalence reported as high as 1 in 700.^3^ Different forms of AI exist. Insufficient or absent matrix secretion causes hypoplastic AI with thin and variably mineralised enamel. Defects during the maturation stage result in hypomineralised AI, where enamel is of full thickness but weak. AI may occur in isolation or as part of syndromic conditions.

Variants in many genes have been implicated in both non-syndromic and syndromic AI to date. These include genes encoding enamel matrix proteins (*AMELX* (MIM*300391), *AMBN* (MIM*610259) and *ENAM* (MIM*606585)), enamel proteases (*MMP20* (MIM*604629) and *KLK4* (MIM*603767)), cell adhesion proteins (*LAMA3* (MIM*600805), *LAMB3* (MIM*150310), *COL17A1* (MIM*113811), *FAM83H* (MIM*611927) and *ITGB6* (MIM*147558)), endocytosis proteins (*WDR72* (MIM*613214)), calcium transport proteins (*SLC24A4* (MIM*609840)) and pH sensors (*GPR68* (MIM*601404)).^4^ In addition, variants have been identified in *C4orf26* (MIM*614829), *AMTN* (MIM*610912)^5^ and *FAM20A* (MIM*611062), encoding proteins for which functions are less clear.

Here we report the identification of two different homozygous missense variants in testicular acid phosphatase *(ACPT*; MIM *606362) causing recessive hypoplastic AI in two families.

## Methods

See Supplementary Information.

## Results

**Figure 1:**
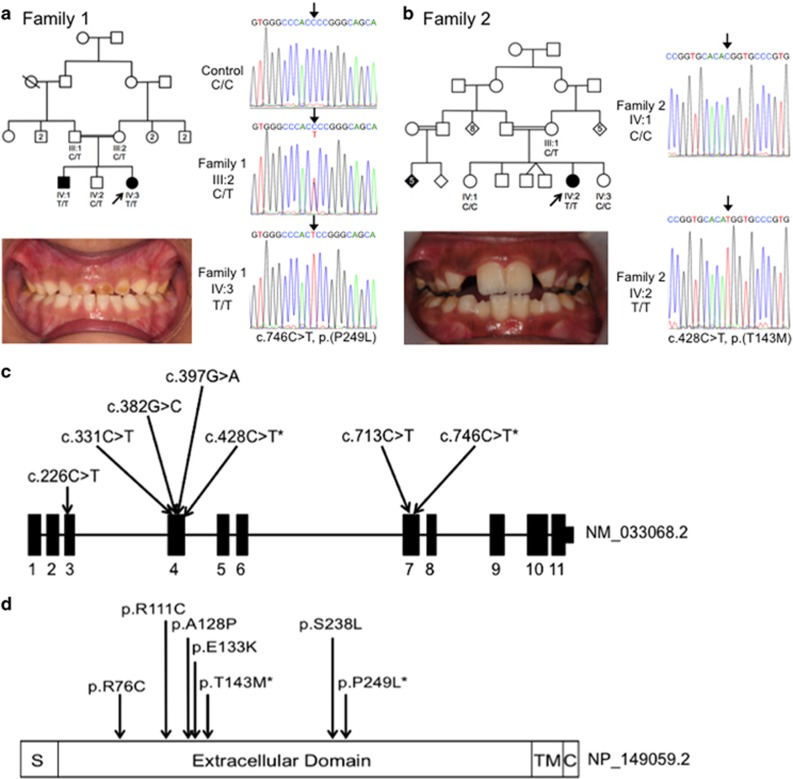
Pedigree, clinical images and variant sequencing traces for families 1 and 2, and the positions of all variants identified in *ACPT* in individuals with AI. Family 1 (**a**) clinical image of IV:2 shows rough, hard hypoplastic AI in the primary dentition. Additional clinical images and radiographs are shown in Supplementary Figure S2. Family 2 (**b**) clinical image of IV:1 shows rough, hard, hypoplastic AI in the mixed dentition. Additional clinical images and radiographs are shown in Supplementary Figure S2. Sequencing traces are shown for the *ACPT* variants identified in family 1: c.746C>T, p.(P249L) and family 2: c.428C>T, p.(T143M). *ACPT* Refseq transcript NM_033068.2, ACPT Refseq protein NP_149059.1. (**c**) Exon and intron structure of *ACPT* (NM_033068.2) labelled with all of the reported variants identified in AI patients. (**d**) Protein domain structure of ACPT (NP_149059.2) labelled with the positions of the residues affected by the variants identified in AI patients. * indicates variants identified in this study. Other variants reported by Seymen *et al.*

Two consanguineous UK Pakistani families segregating autosomal recessive rough, but hard hypoplastic AI (Figure 1; Supplementary Figure S1) were ascertained as part of a cohort of AI families. Neither family had any co-segregating oral or other health problems.

Probands from each family were screened by WES. Mean coverage depth for families 1 and 2 was × 100.1 and × 110.8, respectively (Supplementary Table S2).^6, 7^ As both probands were from first cousin unions, only biallelic, homozygous variants within autozygous regions (Supplementary Tables S3 and S4) were analysed. For both families, three variants remained for each after filtering that segregated with disease in available family members (Supplementary Tables S5 and S6). None of these genes had been previously associated with an AI phenotype. Given the shared AI phenotype, it was notable that both families segregated homozygous missense variants in the gene encoding ACPT.

Family 1 had a homozygous c.746C>T (NM_033068.2) variant in exon 7 predicted to lead to a missense change, p.(P249L) (NP_149059.1). In family 2, a homozygous c.428C>T variant was identified in exon 4, also predicted to lead to a missense change p.(T143M). Sanger sequencing confirmed the *ACPT* variants segregate with the disease phenotype in all available family members (Figure 1).

Both *ACPT* variants are predicted to affect function by a number of different algorithms (Table 1). The c.746C>T, p.(P249L) variant is absent from databases of variation, including dbSNP146,^8^ the Exome Variant Server (http://evs.gs.washington.edu/EVS/) and the ExAC database (v0.3). The c.428C>T, p.(T143M) variant was identified in ExAC in 9 out of 89 042 alleles and has been assigned rs546603773 with 0.02% MAF in dbSNP146 (Supplementary Table S7). The variant was not reported in either database in a homozygous state.

Comparison of ACPT protein sequences in mammals, and of related human proteins lysosomal acid phosphatase 2 (ACP2; MIM*171650) and prostatic acid phosphatase (ACPP; MIM*171790) (Supplementary Figure S3), reveals that the T143 residue is conserved in paralogues and orthologues in all species analysed, except for horse in which the region surrounding T143 is not present. Residue P249 is conserved in orthologues but not in paralogous sequences. Both residues are in the extracellular domain (residues 29–390) of ACPT.^9^ The substitution of threonine at residue 143 for methionine will alter the residue from a small polar to a larger non-polar one (BLOSUM62 score −1). P249 is predicted to form a twist between two alpha helices (Swissmodel Q9BZG2), and substitution of a rigid proline at position 249 with the much larger leucine (BLOSUM62 score −3) is likely to influence the structure of the active site by disrupting that twist.

**Figure 2:**
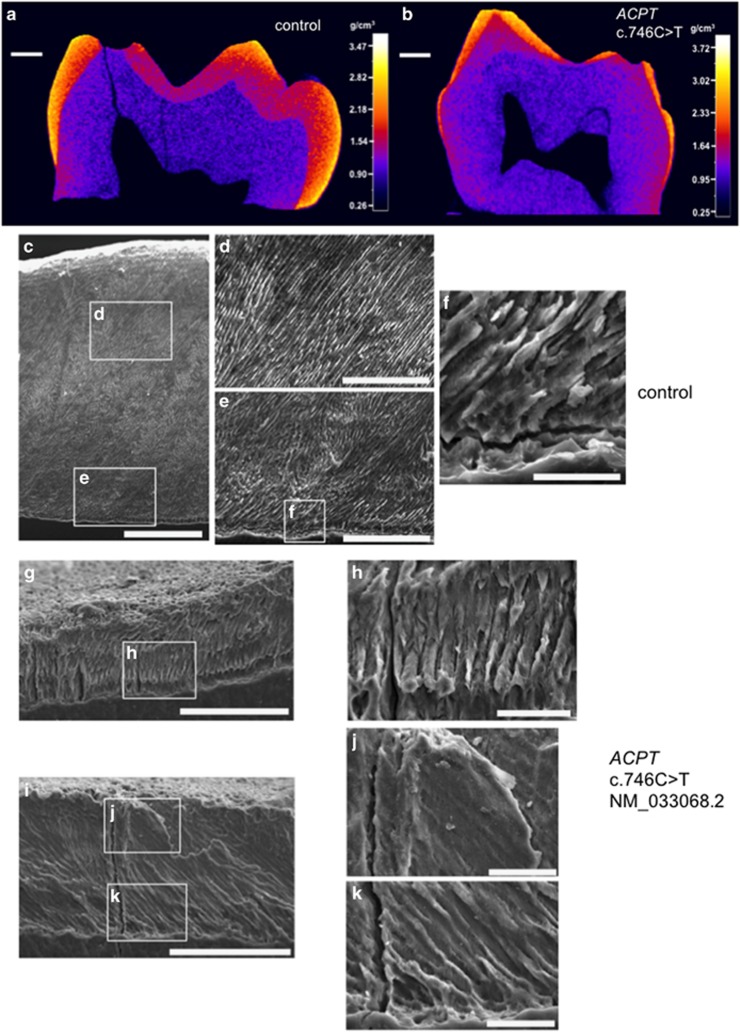
CT and SEM analysis of enamel of a tooth from individual IV:3, family 1 and a control. (**a**) and (**b**) False colour plots to show the variation in enamel mineral density for a cross section through each primary molar tooth. (**a**) control primary molar that has undergone natural exfoliation. Enamel is generally thick but variably mineralised, with the surface enamel being the most mineralised and the EDJ the least. There is a clear demarcation between the enamel and the dentine (**b**) primary molar tooth from individual IV:3, family 1 that was extracted in clinic. The enamel is generally thin but is mineralised where present. The demarcation between the enamel and the dentine is less distinct than for the WT tooth. (**c**–**k**) SEM images of enamel from a control and individual IV:3 (family 1) carrying the homozygous *ACPT* variant, c.746C>T, p.(P249L) based on NM_033068.2 NP_149059.1. (**c**–**f**) Control enamel. (**c**) Entire enamel layer is around 800 *μ*m thick. (**d**) and (**e**) Images of the control enamel show that the typical prismatic architecture is present. (**f**) The enamel at the EDJ. (**g–k**) Individual IV:3 (family 1) enamel. (**g**) and (**i**) Entire enamel layer is around 80–110 *μ*m thick. (**h**) and (**k**) Enamel at the EDJ and around 50 *μ*m into the enamel layer consists of prismatic-like architecture. This architecture is less defined towards the surface (**h**). White boxes show positions of magnified images. Scale bars: (**a**) and (**b**) 1 mm; (**c**) 200 *μ*m; (**d**), (**e**), (**g**) and (**i**) 100 *μ*m; (**f**), (**h**), (**j**) and (**k**) 20 *μ*m.

A primary molar tooth from individual IV:3, family 1, was made available after extraction in clinic (Supplementary Figure S4) and was analysed along with a control primary molar tooth that had undergone natural exfoliation (Figure 2; Supplementary Videos 1 and 2). Analysis by microcomputerised tomography (CT) showed that control enamel varied in its mineral density with a general trend of decrease from the surface enamel to the enamel–dentine junction (EDJ). The enamel layer from the tooth from individual IV:3 was thinner in comparison to the control. At a cusp, where the enamel was at its thickest, a similar trend in enamel mineral density variation to that seen for the control was evident. The enamel present in the tooth from individual IV:3 was mineralised, up to a maximum mineral density of ~3 g/cm^3^ and was comparable to control enamel. The underlying dentine of the tooth from individual IV:3 was mildly hypermineralised with respect to the dentine of the control tooth. Scanning electron microscopy (SEM) showed that control enamel consisted of the typical prismatic architecture (Figures 2c–f). SEM confirmed that the enamel from individual IV:3 was thin (around 80–110 *μ*m) compared to that of the control tooth (around 800 *μ*m). The enamel that was present for the tooth from individual IV:3 appeared to have a prismatic-like architecture, which was more defined nearer to the EDJ (Figures 2g–k).

## Discussion

Here we report the identification of missense variants in the gene encoding the acid phosphatase ACPT, causing autosomal recessive AI in two consanguineous Pakistani families. We also examine the resulting enamel phenotype.

The *ACPT* variants identified are predicted to affect function and segregate with the phenotype in all available family members. The phenotype observed was rough, hard hypoplastic AI in both families, further supporting *ACPT* variants as a cause of AI. These independently made findings confirm the results of Seymen *et al.*,^9^ who recently reported five different *ACPT* missense variants in six Turkish families with a similar recessively inherited hypoplastic AI phenotype. The seven AI-causing *ACPT* variants reported to date are all missense variants within the extracellular domain (residues 29–390) of this 426 amino-acid protein and all give rise to the same distinctive form of AI.

Examination of an extracted primary molar tooth by CT and SEM revealed that the enamel was hypoplastic but similarly mineralised in comparison to control enamel. ACPT is one of a group of enzymes capable of hydrolysing esters of orthophosphoric acid in acidic conditions.^10^ It is expressed by secretory, but not maturation stage ameloblasts,^9^ but the function of ACPT during amelogenesis remains unclear. It has been suggested that it elicits odontoblast differentiation and mineralisation by supplying phosphate during dentine formation.^11^ It is also expressed in the brain, where it may be important for neuronal development and synaptic plasticity.^12^ Examination of the tooth from family 1 individual IV:3 showed that the dentine appeared to be hypermineralised in comparison to that of the control tooth, although no dentine abnormalities were identified upon clinical examination and intraoral dental radiographs. There was also no clinical information to suggest bone involvement, although no formal assessment of the bone phenotype was made. Further analysis of other teeth from individuals with homozygous variants in *ACPT* will be required to assess whether the enamel and dentine findings observed through CT and SEM analysis are typical for individuals with homozygous *ACPT* variants. There was also no clinical information to suggest bone involvement, although no formal assessment of the bone phenotype was made.

The finding that defects in ACPT cause hypoplastic AI is consistent with an essential role for ACPT in the secretory phase of amelogenesis. It is perhaps surprising, given its important role in other tissues, that ACPT defects do not cause syndromic disease. The fact that all *ACPT* AI variants reported to date are missense variants (Figure 1) may suggest loss of a specific function uniquely required by ameloblasts and associated with the extracellular domain of ACPT, or a disease mechanism to which ameloblasts are particularly susceptible. It remains possible that a different phenotype or mode of inheritance is associated with nonsense variants in *ACPT*, or with missense variants affecting other ACPT domains. Interrogation of the ExAC database for variants predicted to lead to loss of ACPT function showed that only heterozygous individuals had been identified for each of the 22 variants observed. This may suggest that ACPT has other important functions and that while missense variants result in AI, homozygous loss of function may be embryonic lethal.

ACPT may be involved in switching odontoblast proliferation to differentiation via beta catenin signalling inhibition, and in dentine mineralisation via production of phosphate and activation of tissue non-specific alkaline phosphatase.^11^ Choi *et al.*^11^ utilised an antibody to block extracellular ACPT domain function and found that odontoblast differentiation was inhibited but proliferation was unaffected. There are no current data to suggest dentinogenesis is significantly affected with homozygous *ACPT* variants.

In summary, we independently discover that missense variants in the extracellular domain of *ACPT* cause rough, hard hypoplastic AI, demonstrating an essential role for this protein in the secretory phase of amelogenesis.

## Figures and Tables

**Table 1:** Pathogenicity scores for the identified *ACPT* variants

*Genomic variant (GRCh37)*	*Transcript variant*	*Predicted amino-acid change*	*SIFT*	*Polyphen-2*	*Mutation Taster*	*CADD v1.3*	*Grantham score*
chr19: g.51297041 C>T	c.746C>T	p.(P249L)	Damaging (0.029)	Probably damaging (0.871)	Disease causing (1.000)	25.4	98
chr19: g.51295037 C>T	c.428C>T	p.(T143M)	Damaging (0.001)	Probably damaging (0.995)	Disease causing (1.000)	27.5	81

Abbreviation: AI, amelogenesis imperfecta. Summary of bioinformatics analyses undertaken to predict the pathogenic nature of the *ACPT* variants identified in families with AI. For each variant, its predicted pathogenic effect and conservation are calculated using a variety of pathogenicity prediction and conservation score software. SIFT and Mutation Taster annotations were based on the relevant Ensembl transcript, Polyphen-2 annotations were based on the relevant RefSeq protein. CADD (v1.3) and Grantham scores are also presented. *ACPT* Ensembl transcript: ENST00000270593 or ACPT RefSeq protein NP_114059.2.
