# Supplementary material for: Defects in the acid phosphatase ACPT cause recessive hypoplastic amelogenesis imperfecta
Source: Eur J Hum Genet. 2017 May 17;25(8):1015–9. doi: 10.1038/ejhg.2017.79 (PMC5511509; doi:10.1038/ejhg.2017.79)
Supplement: Supplementary Methods [file ejhg201779x1.docx]

**Supplementary Methods**

Genomic DNA was obtained from saliva using Oragene^®^ DNA Sample Collection kits (DNA Genotek, Ottawa, ON, Canada). 200ng DNA was prepared for whole-exome sequencing (WES) using the SureSelect All Exon v5 XT reagent (Agilent Technologies, Santa Clara, CA, USA). Sequencing was performed on an Illumina Hi-Seq 3000 sequencing platform (Illumina, San Diego, CA, USA), using a 150bp paired-end protocol. Sequences were aligned to the human reference genome (GRCh37) using BWA.[^1^](#_ENREF_1) The resulting alignment was processed according to GATK best practices.[^2^](#_ENREF_2)

Indel and single-nucleotide variants were called in the VCF format using the Haplotype Caller function of GATK. Autozygosity mapping was carried out on WES data using the program SNPviewer (<https://sourceforge.net/projects/snpviewer/>). Unfiltered VCF files were converted to SNPviewer files using the VCFhacks package (<https://github.com/gantzgraf/vcfhacks>). Autozygous regions >1Mb containing >25 SNPs were highlighted. Variants within autozygous regions, with a minor allele frequency (MAF) <1% in dbSNP142 and the Exome Aggregation Consortium database (ExAC; v0.3; <http://exac.broadinstitute.org/>) and a CADD pathogenicity score of >15 were retained. The remaining variants were annotated using the VCFhacks package.

Variants were confirmed and segregation tested in available family members. Primer sequences for the *ACPT* variants are in Table S1. PCR mastermix HotShot Diamond (Clent Life Science, Stourbridge, UK) was used to amplify sequences. Sanger sequencing was performed using the BigDye Terminator v3.1 kit (Life Technologies, Carlsbad, CA, USA) and resolved on an ABI3130xl sequencer (Life Technologies). Results were analysed using SeqScape v2.5 (Life Technologies).

The *ACPT* variants identified in this study have been submitted to the Leeds AI Leiden Open Variant Database (<http://dna2.leeds.ac.uk/LOVD/> variant ID: #0000000186 and #0000000187) and to ClinVar (accession numbers SCV000494662 and SCV000494663).

A tooth from individual IV:3 (family 1) was analysed by high resolution microCT using a Skyscan 1172 (Bruker, Coventry, UK) operated at 100 kV with a source current of 100 µA and an aluminium / copper filter to reduce beam hardening. CT slices were reconstructed using Skyscan Recon software (Bruker). Calibrated colour contour maps of mineral density were generated using ImageJ and the 3D interactive surface plot plugin. The CT images were calibrated using a two point standard of hydroxyapatite mineral of known densities (0.25 and 0.75 g/cm^3^ (Bruker)). A matched control tooth was obtained from the Skeletal Tissues Research Tissue Bank (School of Dentistry, University of Leeds; NRES REC ref: 07/H1306/95+5). This was obtained with written consent from a patient attending a clinic at Leeds Dental Hospital. CT images were converted into videos using CTVox software (Bruker).

Teeth were sectioned in similar planes using an Accutom-5 cutter (Struers, Ballerup, Denmark) fitted with a peripheral diamond cutting disc and cooled with minimal water.

After sectioning, the cut edge of the tooth was polished using 600 and 2000 grade carborundum paper (3M, Maplewood, MN, USA), followed by a nail buffer. Sections were etched by immersion in 30 % phosphoric acid for 20 s, followed by thorough rinsing in excess distilled water and dried overnight under vacuum. Sections were mounted on aluminium stubs and sputter coated with gold using an auto sputter coater (Agar Scientific, Elektron Technology, Stansted, UK). Microstructural analysis was undertaken using a Hitachi S-3400N scanning electron microscope (Hitachi, Tokyo, Japan), fitted with a 123 eV Nano XFlash® Detector 5010 (Bruker) and operated at an accelerating voltage of 20 kV using secondary electron detection.

**References**

1 Li H, Durbin R. Fast and accurate short read alignment with Burrows-Wheeler transform. *Bioinformatics* 2009; **25**: 1754-1760.

2 Van der Auwera GA, Carneiro MO, Hartl C *et al.* From FastQ data to high confidence variant calls: the Genome Analysis Toolkit best practices pipeline. *Curr Protoc Bioinformatics* 2013; **43**: 11 10 11-33.
