# Supplementary material for: Defects in the acid phosphatase ACPT cause recessive hypoplastic amelogenesis imperfecta
Source: Eur J Hum Genet. 2017 May 17;25(8):1015–9. doi: 10.1038/ejhg.2017.79 (PMC5511509; doi:10.1038/ejhg.2017.79)
Supplement: Supplementary Tables and Figures [file ejhg201779x2.docx]

**Supplementary Tables and Figures**

| **Gene** | **Exon** | **Forward primer (5′-3′)** | **Reverse primer (5′-3′)** | **Size (bp)** |
| --- | --- | --- | --- | --- |
| *ACPT* | 4 | AAGTGAATCTGAGGCTTCTGATTT | TGTCGCAGTTTCTCCCAGAT | 412 |
| *ACPT* | 7 | TTCCCAGCCAGATCCAGATC | CGGGAGAAGTTTGCAAGGAT | 364 |

**Table S1**: **Primer sequences for Sanger sequencing of *ACPT* variants.**


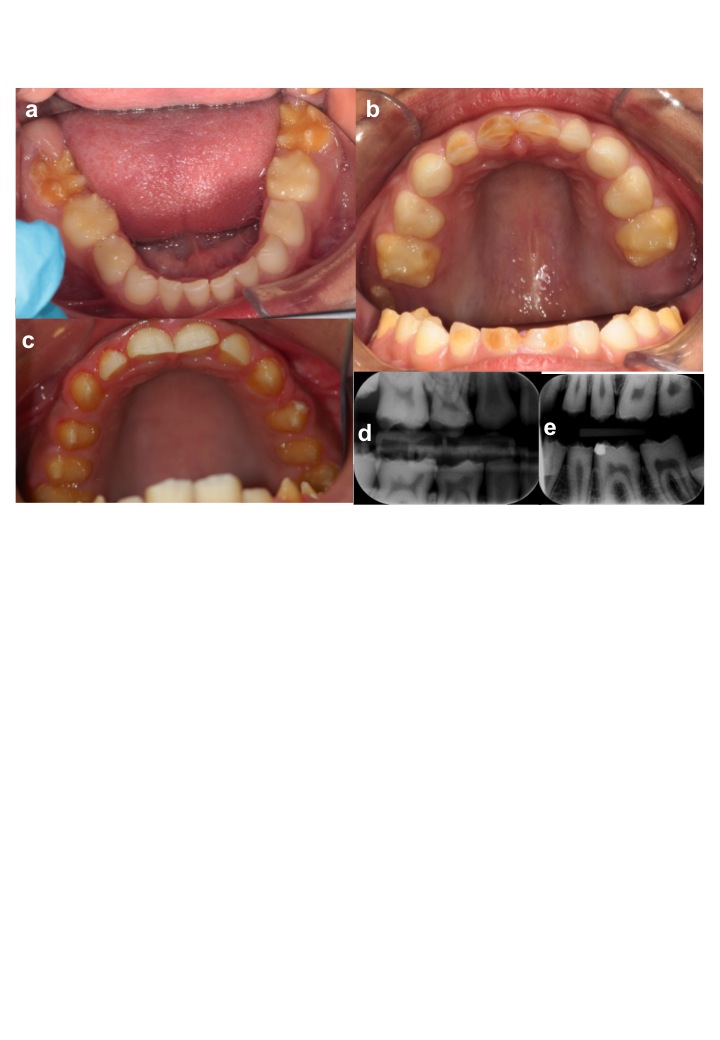


**Figure S1: Additional clinical images for family 1.**

(a) and (b) Clinical images IV:3 showing rough, hard hypoplastic AI in the mixed dentition. An exaggerated cusp architecture is evident for the permanent first molars reflecting the thin enamel overlying the dentine. (c) Clinical image for IV:1 of the permanent dentition. (d) and (e) representative bitewing radiographs for IV:3 (d) and IV:1 (e) confirming a thin layer of well-mineralised enamel. Dentine morphology is within normal limits.


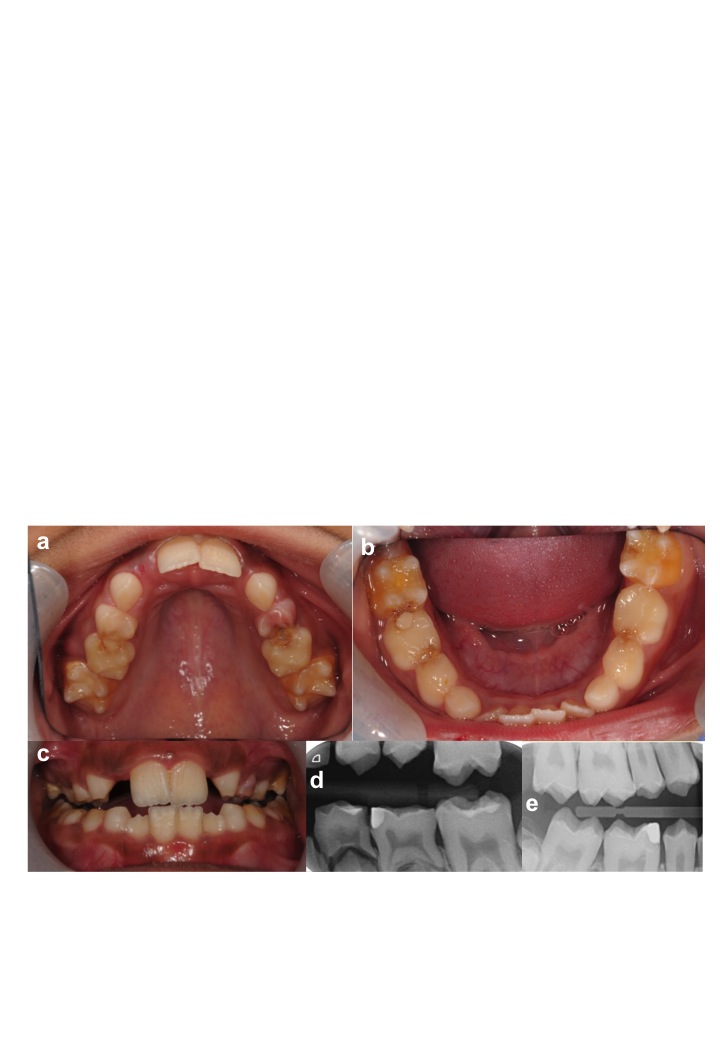


**Figure S2: Additional clinical images for family 2.**

(a) to (c) Clinical images of IV:2 showing a similar phenotype to that observed in family 1. (d) and (e) representative bitewing radiographs for IV:2 confirming that the dentine morphology is within normal limits.

| **Family** | **Sample** | **Mean Coverage** | **% Coverage** | | | | | | **Total reads** |
| --- | --- | --- | --- | --- | --- | --- | --- | --- | --- |
|  |  |  | >4 | >9 | >14 | >19 | >24 | >29 |  |
| 1 | IV:3 | 100.13 | 99.6 | 99.1 | 98.3 | 97.2 | 95.5 | 93.3 | 5042823762 |
| 2 | IV:2 | 110.77 | 99.6 | 99.2 | 98.4 | 97.4 | 96.0 | 94.2 | 5578550261 |

Table S2: Alignment statistics for whole exome sequencing.

Alignment statistics were generated using the regions targetted by the Agilent SureSelectXT Human All Exon V5 capture reagent as the interval.

| **Coordinates** | **rsIDs** | **Size (Mb)** |
| --- | --- | --- |
| chr2:127839434-166535918 | 2_127839434_C/T;2_166535918_C/T | 38.696484 |
| chr5:139060486-156390297 | 5_139060486_G/A;5_156390297_T/C | 17.329811 |
| chr22:33700148-47059939 | 22_33700148_T/C;22_47059939_G/A | 13.359791 |
| chr10:64239709-77312183 | 10_64239709_A/G;10_77312183_TC/T | 13.072474 |
| chr2:272926-10563236 | 2_272926_A/G;2_10563236_G/A | 10.29031 |
| chr5:107197502-117329085 | 5_107197502_G/A;5_117329085_C/T | 10.131583 |
| chr3:45077123-54880461 | 3_45077123_G/A;3_54880461_A/G | 9.803338 |
| chr19:47291174-56410222 | 19_47291174_G/C;19_56410222_C/T | 9.119048 |
| chr2:70488470-79253987 | 2_70488470_C/T;2_79253987_ATAGGGGAAAGT/A | 8.765517 |
| chr7:140476837-148801091 | 7_140476837_T/C;7_148801091_G/A | 8.324254 |
| chr11:126315024-133788869 | 11_126315024_T/C;11_133788869_A/G | 7.473845 |
| chr11:48388717-55111584 | 11_48388717_T/C;11_55111584_A/T | 6.722867 |
| chr22:16157913-22782394 | 22_16157913_T/G;22_22782394_A/G | 6.624481 |
| chr19:9077902-14522434 | 19_9077902_A/G;19_14522434_GCCTTA/G | 5.444532 |
| chr4:104082349-108868515 | 4_104082349_C/T;4_108868515_T/C | 4.786166 |
| chr1:89733273-94461637 | 1_89733273_T/C;1_94461637_C/G | 4.728364 |
| chr10:23297336-27322306 | 10_23297336_A/T;10_27322306_C/T | 4.02497 |
| chr5:6748659-10239261 | 5_6748659_T/C;5_10239261_G/A | 3.490602 |
| chr19:107550-3434413 | 19_107550_A/G;19_3434413_C/G | 3.326863 |
| chr1:55139741-57111169 | 1_55139741_A/G;1_57111169_C/G | 1.971428 |
| chr16:66801241-68711999 | 16_66801241_CA/C;16_68711999_A/G | 1.910758 |
| chr20:31573528-33451148 | 20_31573528_A/C;20_33451148_G/A | 1.87762 |
| chr17:77111732-78820212 | 17_77111732_G/A;17_78820212_G/A | 1.70848 |
| chr14:101350298-102808330 | 14_101350298_T/C;14_102808330_C/T | 1.458032 |
| chr19:39440966-40719410 | 19_39440966_T/C;19_40719410_C/T | 1.278444 |
| chr5:54275119-55412347 | 5_54275119_A/G;5_55412347_A/G | 1.137228 |
| chr19:36381062-37482765 | 19_36381062_A/G;19_37482765_T/C | 1.101703 |

**Table S3: Regions of homozygosity determined by analysis of WES data for IV:3 (family 1) using SNPviewer.**

Only regions >1Mb with a minimum of 25 SNPs are included. The region that includes *ACPT* is shaded grey.

| **Coordinates** | **rsIDs** | **Size (Mb)** |
| --- | --- | --- |
| chr6:97626374-136582417 | 6_97626374_A/G;6_136582417_G/A | 38.956043 |
| chr17:25972311-61901663 | 17_25972311_C/T;17_61901663_C/T | 35.929352 |
| chr4:6536979-36123024 | 4_6536979_G/A;4_36123024_A/G | 29.586045 |
| chr2:220435375-234160345 | 2_220435375_G/A;2_234160345_T/G | 13.72497 |
| chr14:90866346-102359489 | 14_90866346_C/T;14_102359489_A/G | 11.493143 |
| chr18:34664093-45706779 | 18_34664093_A/G;18_45706779_T/C | 11.042686 |
| chr8:1791638-12809667 | 8_1791638_T/G;8_12809667_A/T | 11.018029 |
| chr2:96619799-107423188 | 2_96619799_A/C;2_107423188_G/A | 10.803389 |
| chr7:39472846-50173777 | 7_39472846_G/A;7_50173777_A/G | 10.700931 |
| chr2:79914570-89512854 | 2_79914570_C/CT;2_89512854_A/C | 9.598284 |
| chr2:131338146-138762843 | 2_131338146_AC/A;2_138762843_G/A | 7.424697 |
| chr3:45814094-51680288 | 3_45814094_G/A;3_51680288_C/T | 5.866194 |
| chr17:15604444-20768788 | 17_15604444_A/G;17_20768788_G/T | 5.164344 |
| chr19:49920805-53926522 | 19_49920805_A/G;19_53926522_G/A | 4.005717 |
| chr10:131506283-135438888 | 10_131506283_C/T;10_135438888_C/T | 3.932605 |
| chr2:1-3391826 | 2_45895_A/G;2_3391826_C/T | 3.391825 |
| chr9:1-2182290 | 9_154795_T/C;9_2182290_G/A | 2.182289 |
| chr19:36605647-38702777 | 19_36605647_T/C;19_38702777_C/T | 2.09713 |
| chr16:4920335-6704749 | 16_4920335_A/G;16_6704749_T/G | 1.784414 |
| chr1:223712863-225477546 | 1_223712863_C/A;1_225477546_A/C | 1.764683 |
| chr3:14513695-16242101 | 3_14513695_G/A;3_16242101_C/T | 1.728406 |
| chr5:54253581-55851872 | 5_54253581_A/G;5_55851872_G/A | 1.598291 |
| chr11:66050712-67564162 | 11_66050712_G/A;11_67564162_A/G | 1.51345 |
| chr15:74709975-76067911 | 15_74709975_G/A;15_76067911_A/G | 1.357936 |
| chr1:109839896-110882444 | 1_109839896_C/T;1_110882444_A/G | 1.042548 |
| chr16:1-1034675 | 16_81779_G/T;16_1034675_C/A | 1.034674 |

**Table S4: Regions of homozygosity determined by analysis of WES data for IV:2 (family 2) using SNP viewer.**

Only regions >1Mb with a minimum of 25 SNPs are included. The region that includes *ACPT* is shaded grey.

| **Genomic variant (GRCh37)** | **dbSNP142**  **reference** | **c.DNA variant and predicted amino acid change** | **Gene** | **CADD v1.3** | **SIFT**[**^1^**](#_ENREF_1) | **Polyphen2**[**^2^**](#_ENREF_2) **(HumVar)** | **Mutation**  **Taster**[**^3^**](#_ENREF_3) | **Ensembl transcript** | **RefSeq**  **protein** |
| --- | --- | --- | --- | --- | --- | --- | --- | --- | --- |
| chr19:  g.51297041C>T | N/A | c.746C>T  p.(P249L) | *ACPT* | 25.4 | Damaging  (0.029) | Possibly damaging (0.871) | Disease causing  (0.999) | ENST  00000270593 | NP_149059.1 |
| chr19:  g.52937310A>C | rs201357778 | c.118A>C  p.(N40H) | *ZNF534* | 22.4 | Damaging  (0.001) | Probably damaging (0.998) | Polymorphism  (0.999) | ENST 00000332323 | NP_001137411.1 |
| chr5:  g.149748331C>T | N/A | c.431C>T  p.(T144I) | *TCOF1* | 15.64 | Damaging  (0.016) | Benign  (0.079) | Polymorphism  (0.999) | ENST  00000323668 | NP_000356.1 |

Table S5: Details of the three genomic variants in IV:3 (family 1) that remained after filtering and segregated with disease in all available family members.

Homozygous variants present in IV:3 (family 1) were selected. These were filtered by removing those with a MAF of 1% or more in dbSNP142 or ExAC[^4^](#_ENREF_4) and by selecting only biallelic homozygous variants within regions of homozygosity. The list is restricted to variants scoring 15 or more when scored with CADD v1.3. The segregation of each variant with the disease phenotype was tested for all available family members. Only variants that segregated with disease are shown. The *ACPT* variant is shaded grey. SIFT[^1^](#_ENREF_1) and Mutation Taster[^3^](#_ENREF_3) annotations were based on Ensembl transcript references, Polyphen-2[^2^](#_ENREF_2) annotations were based on RefSeq transcript references. All scores were obtained 25^th^ August 2016.

| **Genomic variant (GRCh37)** | **dbSNP142**  **reference** | **c.DNA variant and predicted amino acid change** | **Gene** | **CADD v1.3** | **SIFT**[**^1^**](#_ENREF_1) | **Polyphen2**[**^2^**](#_ENREF_2) **(HumVar)** | **Mutation**  **Taster**[**^3^**](#_ENREF_3) | **Ensembl transcript** | **RefSeq**  **protein** |
| --- | --- | --- | --- | --- | --- | --- | --- | --- | --- |
| chr19:  g.51295037C>T | rs546603773 | c.428C>T  p.(T143M) | *ACPT* | 27.5 | Damaging (0.001) | Probably damaging (0.995) | Disease causing (0.999) | ENST  00000270593 | NP_149059.1 |
| chr18:  g.43796363G>T | N/A | c.517G>C  p.(G173C) | *C18orf25* | 26.5 | Damaging (0) | Probably damaging (1) | Disease causing (0.999) | ENST  00000282059 | NP_659492.1 |
| chr14:  g.93760283C>A | rs199599034 | c.1083G>T  p.(K361N) | *BTBD7* | 16.52 | Tolerated (0.134) | Benign (0.419) | Disease causing (0.999) | ENST  00000334746 | NM_001002860.2 |

Table S6: Details of the genomic variants identified by WES in IV:2 (family 2) after filtering for homozygous variants.

Homozygous variants present in IV:2 (family 2) were selected. These were filtered by removing those with a MAF of 1% or more in dbSNP142 or ExAC[^4^](#_ENREF_4) and by selecting only biallelic homozygous variants within regions of homozygosity. The list is restricted to variants scoring 15 or more when scored with CADD v1.3. The segregation of each variant with the disease phenotype was tested for all available family members. Only variants that segregated with disease are shown. The *ACPT* variant is shaded grey. SIFT[^1^](#_ENREF_1) and Mutation Taster[^3^](#_ENREF_3) annotations were based on Ensembl transcript references, Polyphen-2[^2^](#_ENREF_2) annotations were based on RefSeq protein reference (* indicates where an alternative transcript was used to score the variant in Polyphen-2 due the specified transcript not being available).

| **Genomic variant (GRCh37)** | **dbSNP146 (reference; alleles)** | **ExAC**[**^4^**](#_ENREF_4) **v0.3**  **(alleles)** | **EVS** |
| --- | --- | --- | --- |
| chr19:g.51297041C>T | Not present | Not present | Not present |
| chr19:g.51295037C>T | rs546603773;  1 / 5008 (heterozygous) | 9 / 89042  (all heterozygous) | Not present |

**Table S7: *ACPT* variants in publically available databases of variation.**

dbSNP: <http://www.ncbi.nlm.nih.gov/SNP/>

ExAC: http://exac.broadinstitute.org/

EVS: http://evs.gs.washington.edu/EVS/

All scores were obtained 18^th^ August 2016.

**(a)** p.T143M

🡻

Guinea pig 147 EADWRPIPVHTVPVAEDKLLR 167

Mouse 132 ETDWKPIPVHTVPVSEDKLLR 152

Rat 133 EADWKPIPVHTVPVSEDKLLR 153

Horse 131 ESAWQVVPD------------ 139

Elephant 131 ESNWRPIPVHTVPVTEDKLLR 151

Macaque 133 EAHWRPIPVHTVPVAEDKLLR 153

Human 133 EARWRPIPVHTVPVAEDKLLR 153

Gorilla 133 EARWRPIPVHTVPVAEDKLLR 153

Chimpanzee 133 EAHWRPIPVHTVPVAEDKLLR 153

Cat 139 EAAWRPIPVHTVPVTEDKLLR 149

Dog 134 EAAWRPIPVHTVPVTEDKLLR 154

Sheep 130 EATWRPIPVHTVPVTEDKLVR 150

Cow 132 EATWRPIPVHTVPVTEDKLLR 152

Wild Boar 132 EATWQPIPVHTVPVTEDKLLR 152

*: *: :*

ACP2 133 NISWQPIPVHTVPITEDRLLK 153

ACPP 135 ILLWQPIPVHTVPLSEDQLLY 155

*: :*

**(b)** p.P249L

🡻

Guinea pig 253 ALDIRAHVGPPRAAEKAQLTG 273

Mouse 238 ALDIRAHVGPPRAAEKAQLTG 258

Rat 239 ALDIRAHVGPPRAAEKAQLTG 259

Horse 210 ALDIGAHVGPPRAAEKAQLTG 230

Elephant 237 ALDIGAHVGPPRAAEKAQLTG 257

Macaque 239 ALDIGAHVGPPRAAEKAQLTG 259

Human 239 ALDIGAHVGPPRAAEKAQLTG 259

Gorilla 239 ALDIGAHVGPPRAAEKAQLTG 259

Chimpanzee 239 ALDIGAHVGPPRAAEKAQLTG 259

Cat 235 ALDIGAHVGPPRAAEKAQLTG 255

Dog 240 ALDIGAHVGPPRAAEKAQLTG 260

Sheep 238 ALDIGAHVGPPQAAEKAQLSG 258

Cow 238 ALDIGAHVGPPQAAEKAQLSG 258

Wild Boar 238 ALDIGAHVGPPRAAEKAQLTG 258

**** ******:*******:*

ACP2 237 DFSFRFLFGIYQQAEKARLQG 257

ACPP 240 ELSLLSLYGIHKQKEKSRLQG 260

:.: * : **::* *

**Figure S3: Clustal Omega multiple sequence alignment of homologous protein sequences for ACPT.**

The arrows indicate the residues altered by the p.T143M (a) and p.P249L (b) substitutions (NM_033068.2; NP_114059.1). Residues are coloured by Clustal Omega according to their properties: Red (AVFPMILW) small, hydrophobic residues; Magenta (RK) basic residues; Green (STYHCNGQ) hydroxyl, sulphydryl, amine and glycine. An asterisk (*) indicates positions that have a single, fully conserved residue. A colon (:) and full stop (.) indicates conservation between groups of similar properties (high and low similarity respectively), these are assigned by Clustal Omega. These are indicated separately for the orthologous sequences only and for the entire dataset.

ACPT sequences used: Mouse, *Mus musculus* NP_001181963.1; Rat, *Rattus norvegicus* XP_008757591.1; Guinea pig, *Cavia porcellus* XP_013003323.1; Elephant, *Loxodonta africana* XP_003406888.1; Rhesus macaque, *Macaca mulatta* XP_001116150.1; Human, *Homo sapiens* NP_149059.1; Gorilla, *Gorilla gorilla* XP_004061298.1; Chimpanzee, *Pan troglodytes* XP_016792167.1; Cow, *Bos taurus* NP_001193896.1; Sheep, *Ovis aries* XP_014955559.1; Horse *Equus caballus* XP_001917480.1; Wild boar, *Sus scrofa* XP_013854163.1; Cat, *Felis Catus* XP_003997517.1; Dog, *Canis lupus familiaris* XP_541473.2.

Parologous sequences: acid phosphatase 2, lysosomal (ACP2) NP_001601.1; acid phosphatase, prostate (APP) NP_001127666.1.


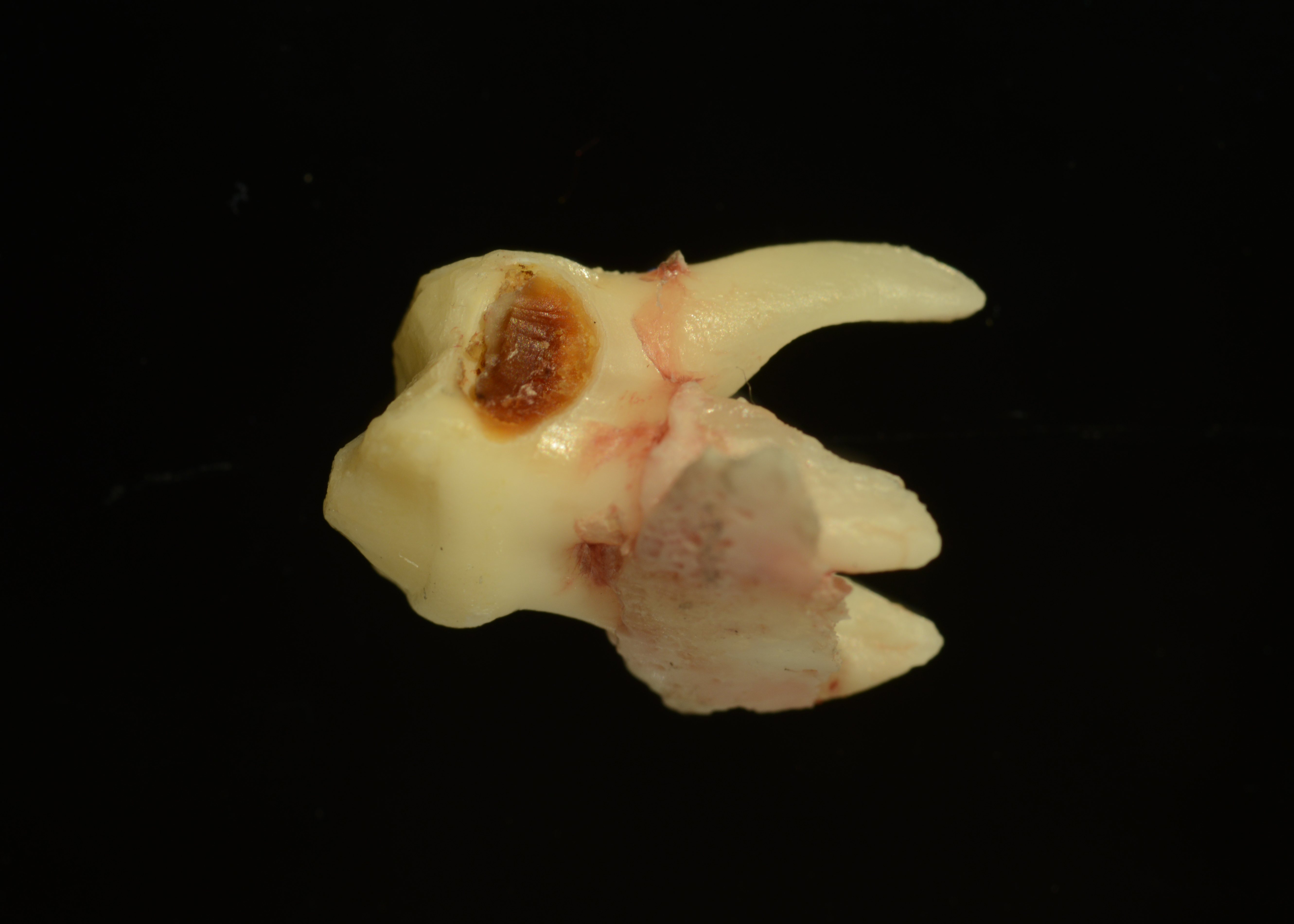


**Figure S4: Deciduous molar tooth made available from individual IV:3, family 1.**

**Supplementary videos:**

Control primary molar tooth

IV:3, family 1 primary molar tooth

**Web Resources**

Combined Annotation Dependent Depletion, http://cadd.gs. washington.edu/info

dbSNP, <http://www.ncbi.nlm.nih.gov/projects/SNP/>

ExAC, <http://exac.broadinstitute.org>

Exome Variant Server, http://evs.gs.washington.edu/EVS/

PolyPhen2 http://genetics.bwh.harvard.edu/pph2/

SIFT, http://sift.jcvi.org/

**References**

1 Ng PC, Henikoff S. SIFT: Predicting amino acid changes that affect protein function. *Nucleic Acids Res* 2003; **31**: 3812-3814.

2 Adzhubei IA, Schmidt S, Peshkin L *et al.* A method and server for predicting damaging missense mutations. *Nat Methods* 2010; **7**: 248-249.

3 Schwarz JM, Rodelsperger C, Schuelke M, Seelow D. MutationTaster evaluates disease-causing potential of sequence alterations. *Nat Methods* 2010; **7**: 575-576.

4 Exome Aggregation Consortium, Lek L, Karczewski K *et al.* Analysis of protein-coding genetic variation in 60,706 humans. *bioRxiv* (2016).
